# Effects of the exercise-inducible myokine irisin on proliferation and malignant properties of ovarian cancer cells through the HIF-1 α signaling pathway

**DOI:** 10.1038/s41598-022-26700-2

**Published:** 2023-01-04

**Authors:** Marziyeh Alizadeh Zarei, Elahe Seyed Hosseini, Hamed Haddad Kashani, Ejaz Ahmad, Hossein Nikzad

**Affiliations:** 1 Gametogenesis Research Center, Institute for Basic Sciences, Kashan University of Medical Science, Kashan, Iran; 2 Anatomical Sciences Research Center, Institute for Basic Sciences, Kashan University of Medical Sciences, Kashan, Iran; 3 Department of Pathology, Michigan Medicine, University of Michigan, Ann Arbor, USA

**Keywords:** Biochemistry, Cancer, Cell biology, Molecular biology, Biomarkers, Oncology

## Abstract

Exercise has been shown to be associated with reduced risk and improving outcomes of several types of cancers. Irisin -a novel exercise-related myokine- has been proposed to exert beneficial effects in metabolic disorders including cancer. No previous studies have investigated whether irisin may regulate malignant characteristics of ovarian cancer cell lines. In the present study, we aimed to explore the effect of irisin on viability and proliferation of ovarian cancer cells which was examined by MTT assay. Then, we evaluated the migratory and invasive abilities of the cells via transwell assays. Moreover, the percentage of apoptosis induction was determined by flow cytometry. Furthermore, the mRNA expression level of genes related to the aerobic respiration (HIF-1α, c-Myc, LDHA, PDK1 and VEGF) was detected by real-time PCR. Our data revealed that irisin treatment significantly attenuated the proliferation, migration and invasion of ovarian cancer cells. Additionally, irisin induced apoptosis in ovarian cancer cells. We also observed that irisin regulated the expression of genes involved in aerobic respiration of ovarian cancer cells. Our results indicated that irisin may play a crucial role in inhibition of cell growth and malignant characteristics of ovarian cancer. These findings may open up avenues for future studies to identify the further therapeutic use of irisin in ovarian cancer management.

## Introduction

Ovarian cancer (OC), as the most common and lethal gynecologic malignancies, is the fifth cause of cancer-related death in women globally^1,2^. Due to the fact that OC is usually asymptomatic at early stages, most of the patients will ultimately progress to advance stages, resulting in a high rate of mortality^3,4^. Despite recent advances, the lack of effective screening methods and therapeutic modalities are the major hurdles to improve prognoses for management of the disease and are the main cause of 5-year survival rate of less than 40%^5^. Thus, exploring novel avenues to improve the status quo is urgently needed.

Obesity has been widely reported to be associated with metabolic diseases including cancer^6–8^. Notably, obese people are usually at increased risk of developing cancers due to the higher levels of insulin and insulin-like growth factor-1, which may be responsible for certain tumors promotion^9^. Exercise is one of the several modifiable factors known to reduce the risk of developing human malignancies^10–13^. It has been also well documented that exercise has many benefits after diagnosis. There is increasingly mounting evidence that exercise relieve many of the common side effects attributed to the conventional cancer therapy among patients, resulting in a better overall quality of life^14–17^. Furthermore, several observational cohort studies support that cancer survivors who had a regular exercise suffered from a lower risk of relapse or cancer-related death after starting treatment regimens^18–20^.

Recently, skeletal muscle is gaining special interest in terms of releasing various myokines which exert beneficial effects in metabolic disorders. Of these, irisin, a novel promising identified myokine released from skeletal muscle following exercise, is believed to be a potential therapeutic in a variety of diseases^21,22^. Previous studies have documented the positive association between irisin and body energy expenditure as well as insulin sensitivity^23^. Additionally, irisin has been verified to have a pivotal role in smooth muscle cell phenotype modulation by regulating endothelial cell proliferation, apoptosis and migration^24^. Due to the strong metabolic effects of irisin on several tissue types, it is questionable whether irisin has the ability to modulate malignant features of cells and tissues similar to other myokines^25^. In the past decade, the relationship between irisin and different cancers has been the focus of many studies. Represented data from a recent study found a lower level of serum irisin in patients with breast cancer, while other studies indicated that irisin was significantly increased in gastrointestinal cancer tissues^26–28^. Moreover, a number of studies have detected increased irisin immunoreactivity in ovarian, cervix and breast cancer tissues as well as endometrial hyperplasia^29^.

In the context of cancer metabolism, reprogramming of glucose metabolism is a pervasive microenvironmental event in tumorigenesis. To this end, cancer cells preferentially switch from oxidative phosphorylation (OXPHOS) to glycolysis, even under normal oxygen concentrations. This particular metabolic profile, defined as Warburg effect, is a key hallmark of many solid tumors including OC^30^.

Growing evidence demonstrated that many signal molecules including tumor suppressor genes and oncogenes play substantial roles in conferring metabolic advantages and adaptation to the tumorigenic microenvironment^30^. Of these documented genes, hypoxia-inducible factor-1α (HIF-1α), is a transcription factor regulating many pivotal pathways in cancerous cells^31^.

Although the importance of HIF-1α in the induction of the Warburg effect is largely highlighted, the involvement and influences of other factors should not be underestimated. A number of studies suggested that crosstalk between HIF-1α and oncogenic c-Myc lead to increased uptake of glucose and its conversion to lactate which subsequently modulate the cancer cell’s microenvironment through regulation of common downstream target genes, such as pyruvate dehydrogenase kinase 1(PDK1) and lactate dehydrogenase A (LDHA)^32^. It is also noticeable that angiogenesis is necessary for tumor growth and metastasis. HIF-1α also upregulates the expression of the angiogenic growth factors such as vascular endothelial growth factor (VEGF), which in turn stimulates neovascularization, a fundamental process for tumor progression^33^.

Considering the aforementioned points, we intended to evaluate the effects of irisin as a promising myokine on tumorigenic features of ovarian cancer cells and to regulate genes correlated with aerobic metabolism.

## Materials and methods

### Cell culture and reagents

The ovarian cancer cell lines OVCAR3, SKOV3 and Caov4 were purchased from the national Cell bank of the Pasteur Institute, Tehran, Iran, were cultured in RPMI 1640 (Gibco, Invitrogen corporation) supplemented with 10% fetal bovine serum (FBS) and 1% penicillin/streptomycin (100 units/mL) and maintained under standard conditions (37 °C and 5% CO_2_). Cells were treated with human recombinant irisin (Cayman Chemical CAS NO: 9037–90-5) at different concentrations ranging from 5 to 70 nM dissolved in phosphate buffer saline (PBS).

### Cell proliferation assay

3-(4,5-Dimethylthiazol-2-yl)-2,5-diphenyltetrazolium bromide (MTT) assay was carried out to determine optimum concentration and time course of action of irisin in OC cell lines (OVCAR3, SKOV3 and Caov4). The mentioned cell lines (10,000 cells/well) in 150 μL medium were seeded overnight into 96-well plates and treated with different doses of irisin (5–70 nM) for different time periods (24, 48, 72, 96 and 120 h). Subsequently, 20 μL of MTT solution (5 mg/mL) was added to each well and incubated for 4 h. Then, 150 μL of dimethyl sulfoxide (DMSO) was added to each and incubated for 15 min at dark. The absorbance at 570 nm was measured using a microplate reader.

### Colony formation assay

OVCAR3, SKOV3 and Caov4 were seeded into a 96-plate at a density of 500 cells/well and treated with 5 and 10 nM of irisin. Untreated cells were considered as controls. All three cell lines were grown for 14 days at standard incubation condition (37 °C and 5% CO_2_). After 14 days of incubation, cells were fixed and stained with 0.5% crystal violet. Colonies including more than 50 cells were attended as surviving cells and colony formation efficiency was calculated as opposed to seeded cells.

### Migration and invasion assays

The in vitro migration and invasion experiments were carried out using a 24-well transwell (BD Bioscience, Bedford, MA). For the invasion test, all cells were serum starved the day before the assay**.** The lower side of the transwell was filled with RPMI 1640 with 10% FBS, and the transwell pre-coated with 50 μL of 1:2 Matrigel/RPMI 1640 (Matrigel, Corning incorporated, USA). All cells (50,000 cells) stimulated with irisin (5 and 10 nM) as well as untreated control cells suspended in serum-free medium and were added to the upper surface of the transwell. After incubation for 48 h in 37 °C, the medium was completely removed from the top part and non-invaded cells were wiped using cotton swabs. The cell invading to the underside of the transwell were fixed and stained in 0.1% crystal violet. The number of invaded cells through the matrigel was enumerated in 5 randomly fields using an inverted microscope (200 ×). The migration assay was conducted with a similar method, except cells were plated into uncoated-matrigel transwell and incubated for 24 h.

### Apoptosis detection

Annexin-V/PI staining kit (Roche Applied Science, Penzberg, Germany) was used to analyze apoptosis percentage in OC cells according to the manufacturer’s instruction. Briefly, OVCAR3, SKOV3 and Caov4cells were seeded (200,000 cells/well) into six-well plates and incubated overnight. At 70–80% confluency, cells treated with desired concentration of irisin (10 nM) and incubated for additional 48 h. After this, cells were trypsinized and washed with PBS twice. Trypsin-digested cells were centrifuged and cells pellet were resuspended in 100 μL of binding buffer and incubated for 15 min at 15–25 °C. Finally, apoptotic cells were analyzed using FACSCalibur flow cytometer within 1 h.

### Real-time quantitative polymerase chain reaction (qPCR)

Total RNA was extracted from treated cells using Trizol (Invitrogen, California, USA). Synthesis of Complementary DNA was performed by PrimeScript™ RT reagent kit (Takara Bio Inc., Shiga, Japan), and flowingly used as template for Real-time PCR reaction which was conducted by IQ5 (Bio-Rad, Germany). The changes in mRNA expression of genes of interest were normalized to the levels of the glyceraldehyde-3-phosphate dehydrogenase (GAPDH) as internal reference. Real-time data analysis performed using 2^−ΔΔct^ formula. The sequences of specific primers are shown in Table 1.

**Table 1:** Primers used in real-time PCR.

Gene	Primers	Amplicon (bp)
HIF-1α	Forward- 5′ AAATGTTCTGCCTACCCTGTTG 3′	152
	Reverse- 5′ GGATGTTAATAGCGACAAAGTGC 3′	
c-Myc	Forward- 5′ ACACATCAGCACAACTACGC 3′	164
	Reverse- 5′ GTTCGCCTCTTGACATTCTCC 3′	
LDHA	Forward- 5′ CTGTATGGAGTGGAATGAATGTTG 3′	160
	Reverse- 5′ ATAGCCCAGGATGTGTAGCC 3′	
PDK1	Forward- 5′ ACCATGTTCAGTTCCTTATCTACC 3′	168
	Reverse- 5′ CTGTCATTTCATCATCTGTGTGC 3′	
VEGF	Forward- 5′ GGCTCCTCTCCAGTCTGATC 3′	200
	Reverse- 5′ AGCTTAGGAACGTGTGGTCA 3′	
MMP2	Forward- 5′ TTGATGGCATCGCTCAGATC 3′	175
	Reverse- 5′ TTGTCACGTGGCGTCACAGT 3′	
MMP9	Forward- 5′ GACGCAGACATCGTCATCCA 3′	190
	Reverse- 5′ CACAACTCGTCATCGTCGAAA 3′	
GAPDH	Forward- 5′ GAGTCAACGGATTTGGTCGT 3′	237
	Reverse- 5′ TTGATTTTGGAGGGATCTCG 3′	

### Statistical analysis

Statistics were presented in Prism® 5 software (GraphPad Software, Inc., La Jolla, CA, USA). The data are expressed as the mean ± standard deviation (SD). All experiments were repeated at least three times. Comparisons between the groups were conducted using one-way ANOVA analysis of variance followed by Tukey’s HSD multiple comparison test. Statistical significance was considered significant at *P* < 0.05.

## Results

### Regulation of cell proliferation by irisin in ovarian cancer cell lines

**Figure 1:**
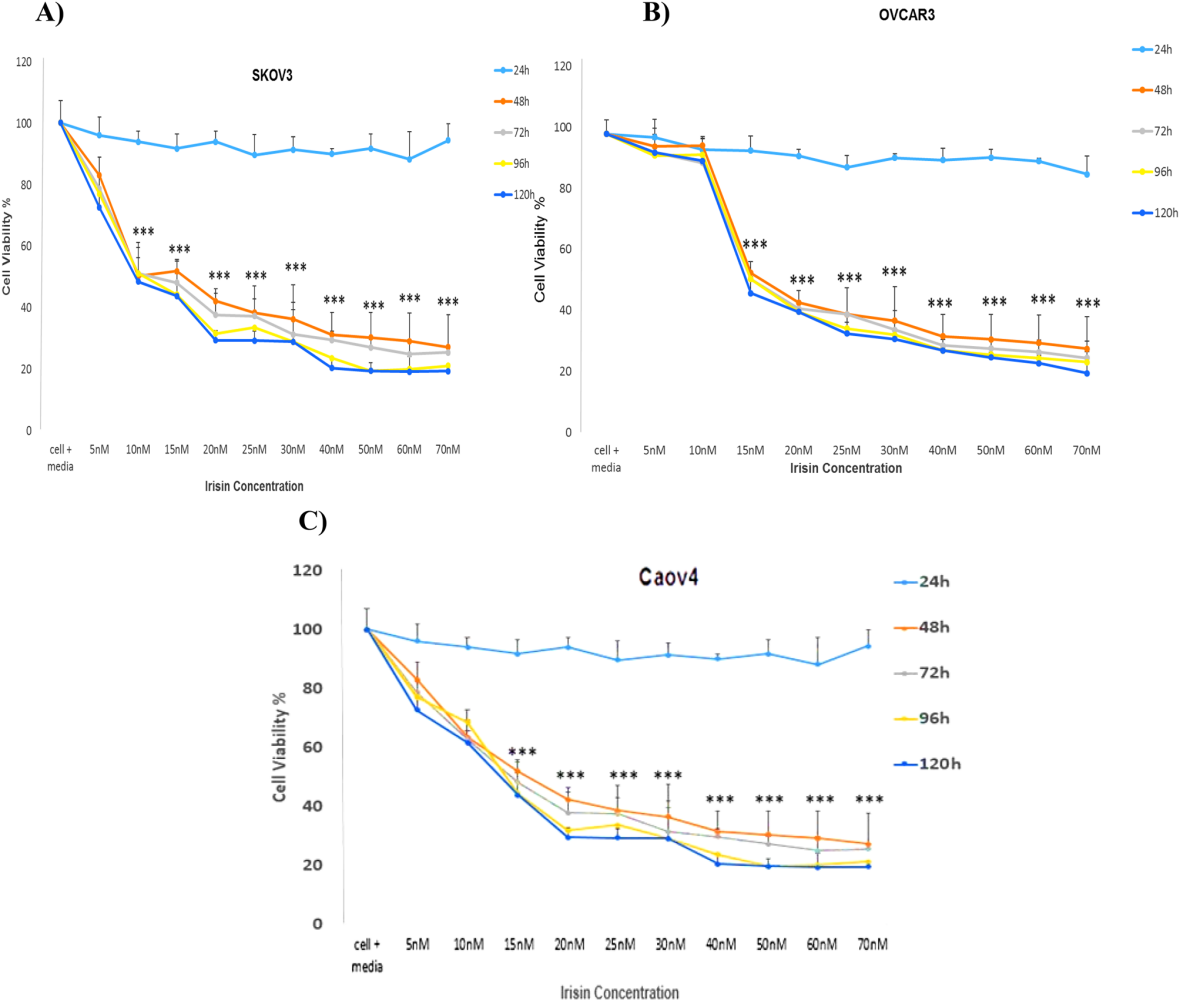
The effect of irisin on the proliferation of **(A)** SKOV3 **(B**) OVCAR3 **(C**) Caov4 cells in 24, 48 and 72, 96 and 120 h. Data expressed as mean ± standard deviation; *P < 0.05; **P < 0.01; ***P < 0.001 compared to no treated cells.

MTT assay was used to investigate the effect of various concentrations of irisin within different time periods on OC cells viability. We found no significant changes in the viability of OC cells 24 h after treatment. However, our data indicated that irisin significantly suppressed the proliferation of all three cell lines in a time- and dose-dependent manner (Fig. 1A–C).

### Effects of irisin on colony formation ability of ovarian cancer cell lines

**Figure 2:**
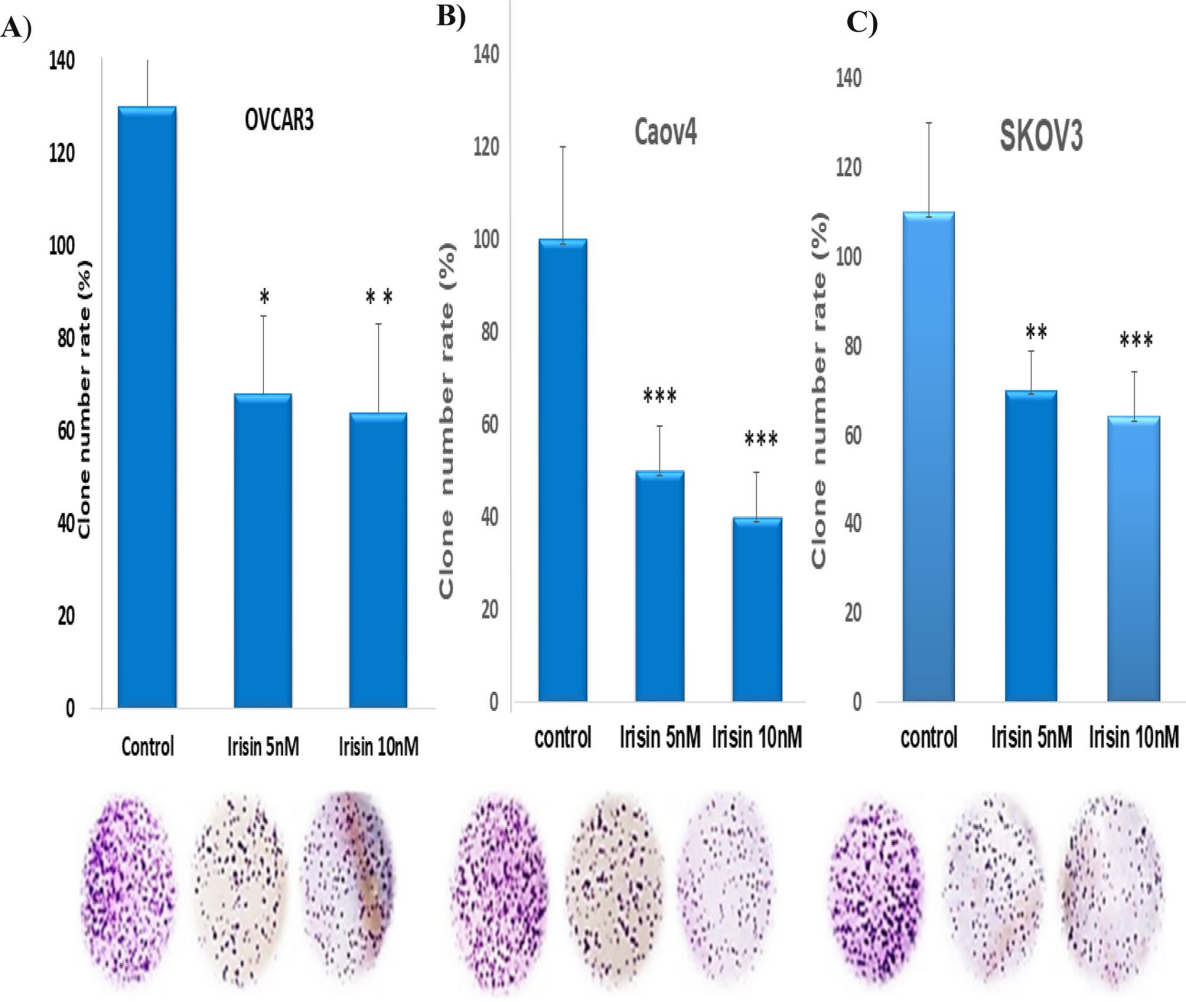
Effect of irisin to inhibit colony formation of **(A)** OVCAR3, (**B)** Caov4 and (**C**) SKOV3 and ovarian cancer cell lines. Data shows the representative of three independent experiments. Data expressed as mean ± standard deviation; *P < 0.05; **P < 0.01; ***P < 0.001 compared to no treated cells.

The ability of three cell lines examined to form colonies on 6-well plates in the presence or absence of irisin (5 and 10 nm) within 2 weeks. Our findings represented that the colonogenic potential of OC cells was significantly suppressed after treatment with irisin. Although, the greatest inhibitory effect of irisin on cologenic ability was observed in Caov4 cells, the number of colonies formed attenuated in a dose dependent manner in all three cell lines tested, as shown in (Fig. 2A–C).

### Effects of irisin on invasiveness properties of ovarian cancer cell lines

**Figure 3:**
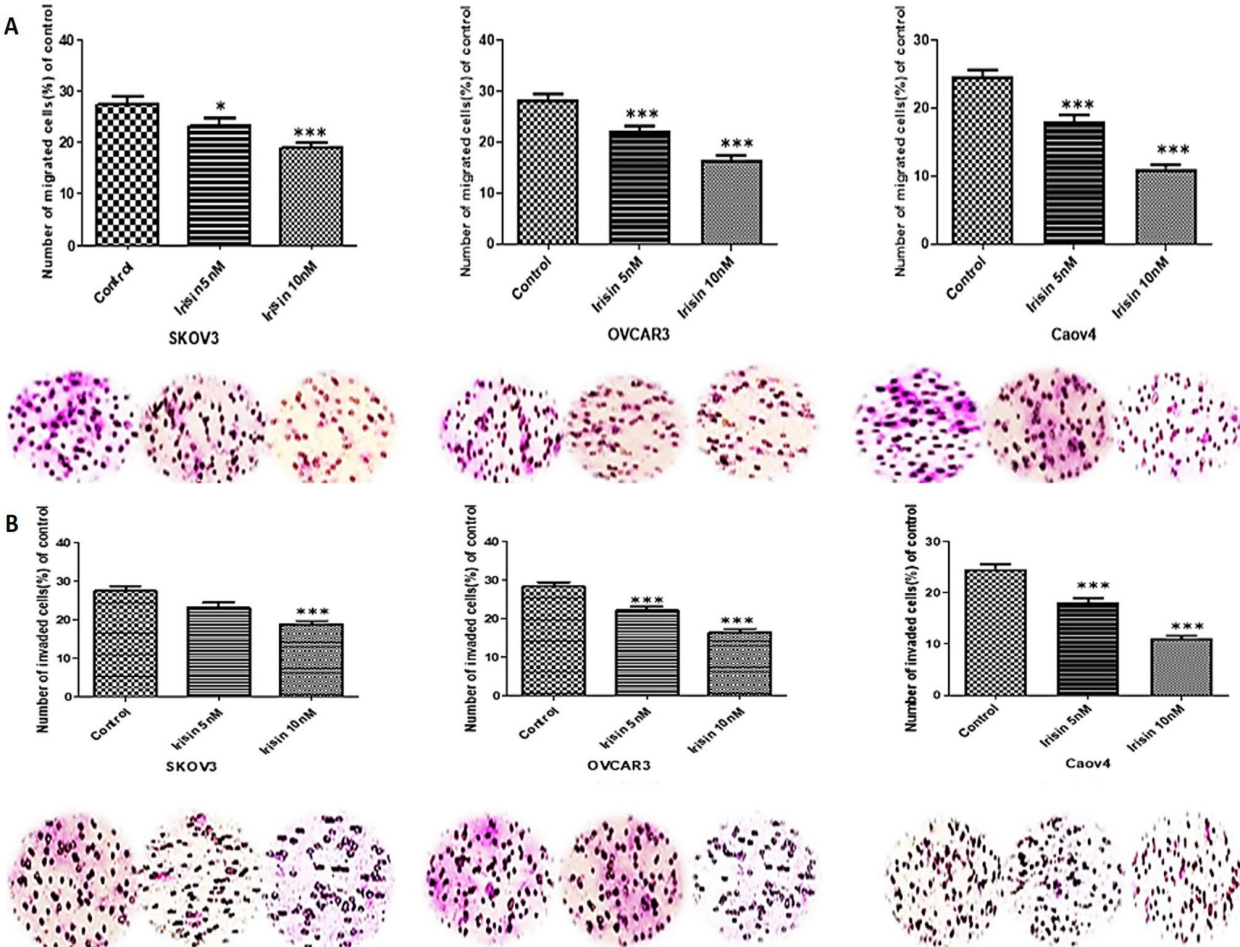
(**A**) The effect of Irisin on the migratory behavior. (**B**) The effect of Irisin on invasiveness of OVCAR3, SKOV3 and Caov4 cells. Data expressed as mean ± standard deviation; **P, 0.01; ***P, 0.001 compared to control.

We applied the trans well system to explore the effect of irisin on the migration and invasion of OC cell lines. As our results revealed, irisin effectively reduced the migratory behavior of all three cell lines as compared to control groups. Furthermore, represented data suggested the inhibitory effect of irisin on invasiveness of OC cell lines. The migration and invasion potential of all cell lines subjected to irisin were decreased in a dose-dependent manner significantly (Fig. 3A, B).

### The effects of irisin on apoptosis induction in ovarian cancer cell lines

**Figure 4:**
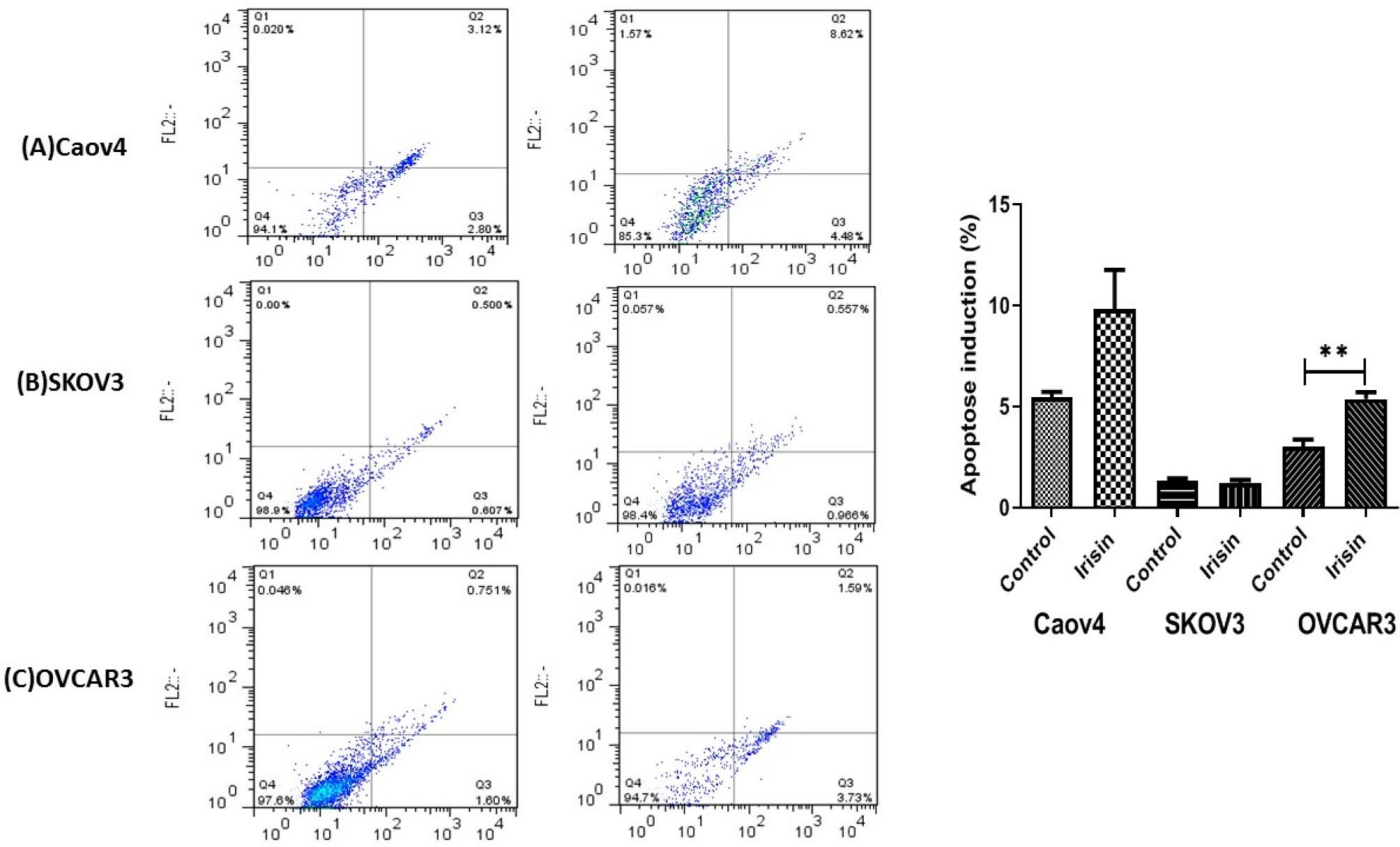
The effect of irisin on apoptosis induction of (**A**) Caov4, (**B**) SKOV3 and (**C**) OVCAR3 in ovarian cancer cells**.** Data expressed as mean ± standard deviation; **P, 0.01 compared to control.

 Given the inhibitory effects of irisin on OC cell proliferation, we next examined the susceptibility of OC cells to apoptosis in the presence 10 nM of irisin. Flow cytometry analysis illustrated that irisin caused a significant rise in the percentage of apoptotic OVCAR3 cells as compared to control cells (*P* < 0.008), however, no significant effect was detected in other two cell lines (Fig. 4A–C).

### Irisin affects the expression of aerobic metabolism markers through the HIF1-α signaling pathway in ovarian cancer cells

**Figure 5:**
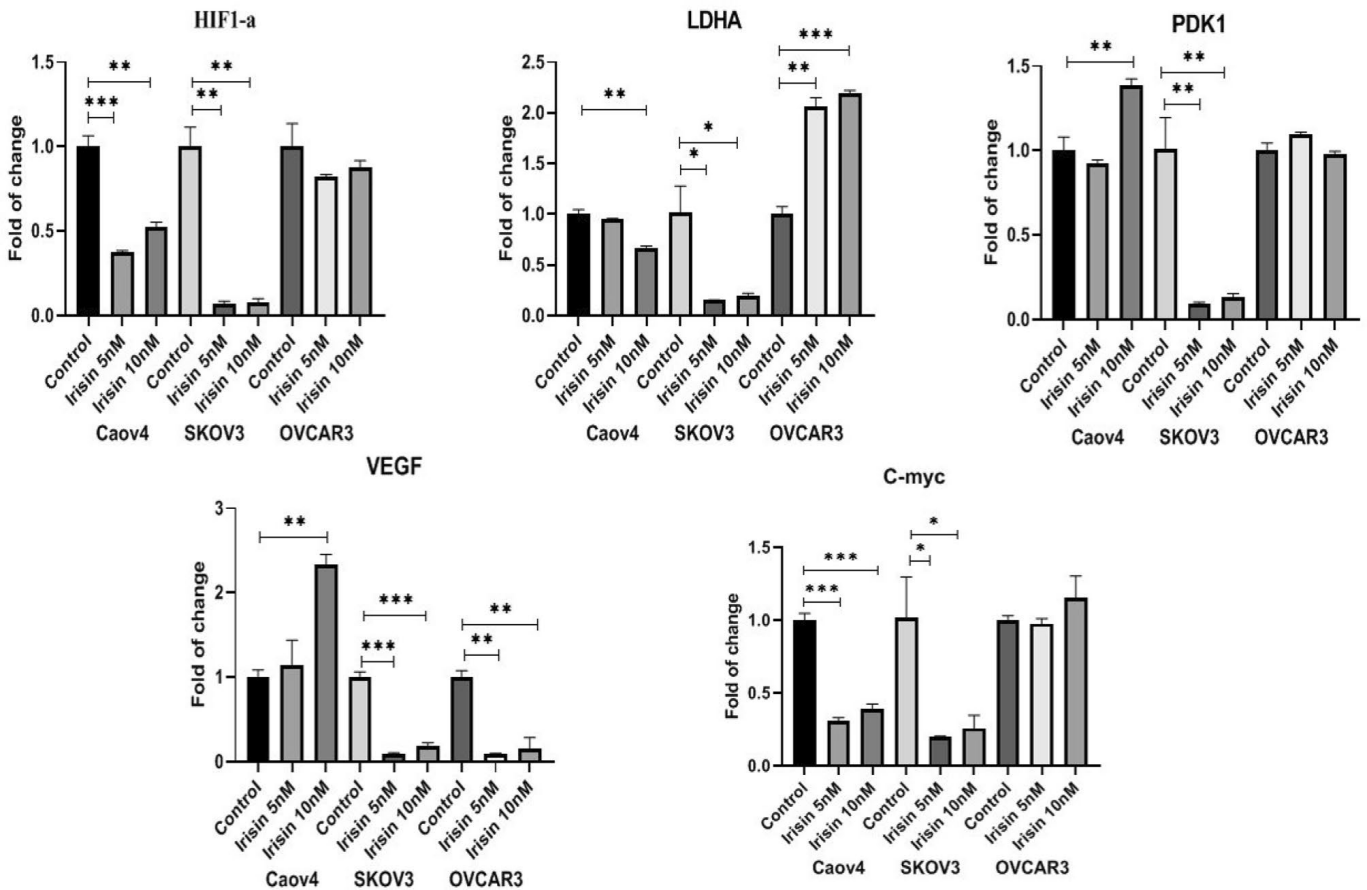
Effect of Irisin on HIF-1α signaling pathway genes 48 h after treatment with 5 nM and 10 nM of irisin. Data expressed as mean ± standard deviation; *P ≤ 0.05 **P ≤ 0.01; ***P ≤ 0.001 compared to control.

The expression of aerobic metabolism genes in OC cells in the absence or presence of irisin (5 and 10 nM) was measured using real-time PCR. As depicted, irisin decreased the expression of HIF-1α, c-Myc and LDHA genes in SKOV3 and Caov4 cells significantly compared to control cells, however PDK1 gene was downregulated only in SKOV3 cells and no significant reduction in the level of mentioned genes was found in OVCAR3 cells. In the case of VEGF expression, irisin attenuated the expression level of this gene in both SKOV3 and OVCAR3 cells. Surprisingly, there was greater inhibitory effect of irisin on VEGF expression observed after treatment with 5 nM of irisin (Fig. 5).

### The effect of irisin on the expression of MMP2 and MMP9 in ovarian cancer cells

**Figure 6:**
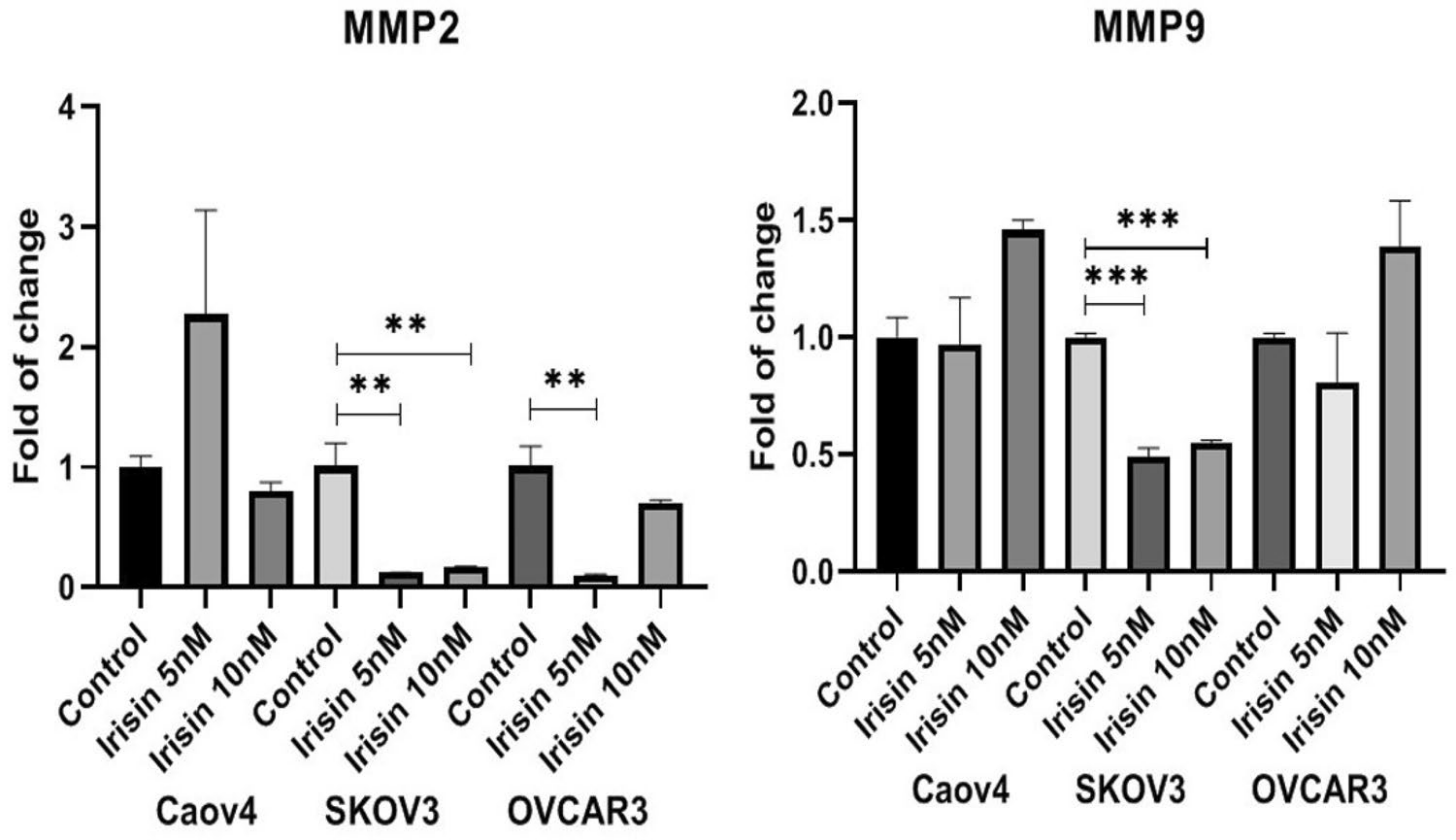
Effect of Irisin on MMP2 and MMP9 expression levels 48 h after treatment with 5 nM and 10 nM of irisin. Data expressed as mean ± standard deviation; **P ≤ 0.01; ***P ≤ 0.001 compared to control.

The expression of MMP2 in OC cells treated with irisin was measured using real-time PCR. The results show irisin remarkably decreased the expression of MMP2 in SKOV3 and OVCAR3 cells while MMP9 downregulated only in SKOV3 cells as compared to control. However, irisin had no significant effect on MMP9 and MMP2 expression in Caov4 and MMP9 in OVCAR3 cells (Fig. 6, Supplementary Information).

## Discussion

Ovarian cancer, as the most frequent gynecologic malignancies, is a type of cancer accompanied with hypoxic microenvironment^34–36^. Exercise is known to be a prominent component of cancer treatment strategies. However, the effects of exercise on tumor oxygenation, hypoxia and vascularization are not fully understood^37^.

The purpose of the current study was to investigate the effects of irisin, as one of the novel exercise-related hormones, on proliferation and malignant facet of OC. We have also provided evidence about the influence of irisin on expression of HIF-1α and its target genes as plausible contributors in driving OC tumorigenesis. We found that irisin suppressed the proliferation and viability of OC cells in a concentration and time-dependent mode. We further checked the effect of irisin on long-term survival of OC cells. Our data showed that irisin caused a significant decline in the colony-forming potential of OC. Several complex processes including cell migration and invasion attributed in tumor dissemination, suggesting the potential therapeutic interventions required for targeting of extracellular matrix degradation^38^. As expected, our results illustrated that irisin significantly mitigated the migratory behavior and invasiveness of all three cell lines as opposed to control groups in a dose-dependent way, representing the anti-metastatic trait of irisin. As matrix metalloproteinases (MMPs) mediate cancer cell invasion and metastasis in human malignancies, including OC^38^, in this study we aimed to explore whether irisin affects the mRNA expression level of MMP2 and MMP9 as two verified metastasis markers in OC. Our results determined that irisin markedly reduced the mRNA expression level of MMP2 in OVCAR3 and SKOV3 cells. However, no significant decrease in the mRNA expression level of MMP9 was detected in surveyed cells after treatment with irisin except in SKOV3 cells. Nevertheless, the intricate mechanisms implicated with MMPs inhibition in OC cells need to be further studied. Pervious findings by Kong et al*.* suggested that irisin reduced the expression of MMP2 and MMP9 in osteosarcoma cells via suppressing IL-6^39^. Since a number of studies displayed a link between the type of exercise and MMPs regulation, the effect of irisin on MMPs expression level in vitro may be different from which occurs in cancer patients^40^. Therefore, it is conceivable to explore the relationship between irisin, as an exercise-derived hormone, and MMPs expression after different types of exercise activities in cancer patients. To test the susceptibility of OC cells to apoptosis, we next examined the percentage of apoptosis of OC cells in the presence of 10 nM irisin. Our analysis showed that irisin significantly enhanced the percentage of apoptotic OVCAR3 cells as compared to control cells (*P* < 0.008), however, apoptosis did not upsurge in other two cell lines. More exhaustive understanding of cancer biology at the cellular and molecular levels have accentuated the HIF-1α pathway as a pivotal pathway in cancer development, for which targeting the HIF-1α pathway could be an emerging area of research^33^. HIF-1α, as the central regulator of oxygen homeostasis, orchestrates adaptive adjustments to hypoxic conditions^41,42^. It has been repeatedly reported that HIF-1α remarkably upregulates in OC or borderline tissues in relative to benign tissues^43,44^. More specially, HIF-1α expression is known to be linked with clinicopathological aspects of OC and patients' outcome including FIGO stage, histological subtype, metastasis and 5-year survival rate^45^. Extensive studies have elucidated that molecular consequences of HIF-1α upregulation are induced expression of numerous genes linked with different aspects of cancer development, including proliferation (MYC), angiogenesis (VEGF), metabolism (PDK1, LDHA) and extracellular matrix disturbance (MMP2, MMP9)^46,47^. In this study, we assessed whether irisin had suppressive effect on expression level of HIF-1α and its target genes in OC cells. Our real-time experiments verified that SKOV3 cells had a lower expression level of genes involved in HIF-1α pathway after exposure to irisin. Meanwhile, no considerable change was detected in OVCAR3 cells after treatment with the same concentration of irsin. HIF-1 activity is induced by mTOR-altered metabolism and induced glycolysis via adaptation to hypoxia. The HIF-1 and HIF-2 heterodimers result in metabolic shift through responding to hypoxia^48^. Of these two heterodimers, HIF-1 is a leading component attributed to tumor metabolism resulting in overexpression of glucose transporters as well as PDK1, an enzyme which hamper pyruvate entrance to the TCA cycle^49^. Qiao et al*.* found that malignant lymphomas represent noteworthy expression of HIF-1α. This expression is mediated through nuclear factor kappa light-chain-enhancer of activated B cells (NF-κB). Notably, radiotherapy of lymphoma exhibited enhanced NF-κB activation and high level of HIF-1α. This reveals that targeted therapy of HIF-1α combined with radiation therapy of lymphoma cells could improve treatment efficacy^50^. The c-Myc proto-oncogene regarded as an eminent regulator in many cellular processes including cell cycle, cell proliferation and apoptosis. c-Myc expression and subsequently LDHA expression have been reported to be downregulated during exercise^51^. One additional striking mechanism is the impact of exercise on growth factors such as (VEGF) as a potent angiogenic stimulator. Dysregulation of VEGF has been long known to be linked with the development of almost all tumors leading to distant metastasis, which is one of the main cause responsible for patient mortality^52,53^.

## Conclusion

Considering the aforementioned points, exercise is a master regulator of many genes contributed to cancer development. Our encouraging results in line with previous studies suggest that irisin, as an exercise-derived hormone, may be a potential anticancer agent, warranting the need for more detailed investigations for having better insights about irisin effects as well as underlying mechanisms in cancer therapy.

## Supplementary Information

Supplementary Information.

## Data Availability

The data that support the findings of this study are available from the corresponding author upon reasonable request.

## Abbreviations

OC: Ovarian cancer
OXPHOS: Oxidative phosphorylation
HIF-1α: Hypoxia-inducible factor-1α
PDK1: Pyruvate dehydrogenase kinase 1
LDHA: Lactate dehydrogenase A
VEGF: Vascular endothelial growth factor
FBS: Fetal bovine serum
PBS: Phosphate buffer saline
MTT: 3-(4,5-Dimethylthiazole-yl)-2, 5-diphenyltetrazolium bromide
DMSO: Dimethylsulfoxide
GAPDH: Glyceraldehyde-3-phosphate dehydrogenase
MMPs: Matrix metalloproteinase
NF-κB: Nuclear factor kappalight- chain-enhancer of activated B

## References

[1] Hosseini ES, Meryet-Figuiere M, Sabzalipoor H, Kashani HH, Nikzad H, Asemi Z (2017). Dysregulated expression of long noncoding RNAs in gynecologic cancers. Mol. Cancer.

[2] Meryet-Figuière M, Lambert B, Gauduchon P, Vigneron N, Brotin E, Poulain L, Denoyelle C (2016). An overview of long non-coding RNAs in ovarian cancers. Oncotarget.

[3] Hosseini ES, Zarei MA, Babashah S, Sistani RN, Sadeghizadeh M, Kashani HH, Mahabadi JA, Izadpanah F, Atlasi MA, Nikzad H (2019). Studies on combination of oxaliplatin and dendrosomal nanocurcumin on proliferation, apoptosis induction, and long non-coding RNA expression in ovarian cancer cells. Cell Biol. Toxicol..

[4] Narod S (2016). Can advanced-stage ovarian cancer be cured?. Nat. Rev. Clin. Oncol..

[5] Heindl A, Khan AM, Rodrigues DN, Eason K, Sadanandam A, Orbegoso C, Punta M, Sottoriva A, Lise S, Banerjee S (2018). Microenvironmental niche divergence shapes BRCA1-dysregulated ovarian cancer morphological plasticity. Nat. Commun..

[6] Birks S, Peeters A, Backholer K, O'Brien P, Brown W (2012). A systematic review of the impact of weight loss on cancer incidence and mortality. Obes. Rev..

[7] Ferguson, R. D., Gallagher, E. J., Scheinman, E. J., Damouni, R. & LeRoith, D. The epidemiology and molecular mechanisms linking obesity, diabetes, and cancer, in *Vitamins and Hormones*. 51–98 (Elsevier, 2013). 10.1016/B978-0-12-416673-8.00010-1 23810003

[8] Tao W, Lagergren J (2013). Clinical management of obese patients with cancer. Nat. Rev. Clin. Oncol..

[9] Matafome P, Santos-Silva D, Sena C, Seica R (2013). Common mechanisms of dysfunctional adipose tissue and obesity-related cancers. Diabetes/Metab. Res. Rev..

[10] Boyle T, Keegel T, Bull F, Heyworth J, Fritschi L (2012). Physical activity and risks of proximal and distal colon cancers: a systematic review and meta-analysis. J. Natl. Cancer Inst..

[11] Keimling M, Behrens G, Schmid D, Jochem C, Leitzmann M (2014). The association between physical activity and bladder cancer: systematic review and meta-analysis. Br. J. Cancer.

[12] Kenfield SA, Batista JL, Jahn JL, Downer MK, Van Blarigan EL, Sesso HD, Giovannucci EL, Stampfer MJ, Chan JM (2015). Development and application of a lifestyle score for prevention of lethal prostate cancer. J. Natl. Cancer Inst..

[13] Wu Y, Zhang D, Kang S (2013). Physical activity and risk of breast cancer: a meta-analysis of prospective studies. Breast Cancer Res. Treat..

[14] Fong DY, Ho JW, Hui BP, Lee AM, Macfarlane DJ, Leung SS, Cerin E, Chan WY, Leung IP, Lam SH (2012). Physical activity for cancer survivors: meta-analysis of randomised controlled trials. BMJ.

[15] Gerritsen JK, Vincent AJ (2016). Exercise improves quality of life in patients with cancer: a systematic review and meta-analysis of randomised controlled trials. Br. J. Sports Med..

[16] Mishra, S. I., Scherer, R. W., Snyder, C., Geigle, P. M., Berlanstein, D. R. & Topaloglu, O. Exercise interventions on health‐related quality of life for people with cancer during active treatment. *Cochrane Database Syst. Rev.* (2012). 10.1002/14651858.CD008465.pub2 PMC7389071 22895974

[17] Tomlinson D, Diorio C, Beyene J, Sung L (2014). Effect of exercise on cancer-related fatigue: a meta-analysis. Am. J. Phys. Med. Rehabil..

[18] Irwin ML, Alvarez-Reeves M, Cadmus L, Mierzejewski E, Mayne ST, Yu H, Chung GG, Jones B, Knobf MT, DiPietro L (2009). Exercise improves body fat, lean mass, and bone mass in breast cancer survivors. Obesity.

[19] Kenfield SA, Stampfer MJ, Giovannucci E, Chan JM (2011). Physical activity and survival after prostate cancer diagnosis in the health professionals follow-up study. J. Clin. Oncol..

[20] Meyerhardt JA, Sato K, Niedzwiecki D, Ye C, Saltz LB, Mayer RJ, Mowat RB, Whittom R, Hantel A, Benson A (2012). Dietary glycemic load and cancer recurrence and survival in patients with stage III colon cancer: findings from CALGB 89803. J. Natl. Cancer Inst..

[21] Boström P, Wu J, Jedrychowski MP, Korde A, Ye L, Lo JC, Rasbach KA, Boström EA, Choi JH, Long JZ (2012). A PGC1-α-dependent myokine that drives brown-fat-like development of white fat and thermogenesis. Nature.

[22] Karstoft K, Pedersen BK (2016). Skeletal muscle as a gene regulatory endocrine organ. Curr. Opin. Clin. Nutr. Metab. Care.

[23] Wu J, Boström P, Sparks LM, Ye L, Choi JH, Giang A-H, Khandekar M, Virtanen KA, Nuutila P, Schaart G (2012). Beige adipocytes are a distinct type of thermogenic fat cell in mouse and human. Cell.

[24] Song H, Xu J, Lv N, Zhang Y, Wu F, Li H, Shao L, Mu Q, Wang F, Tang D (2016). Irisin reverses platelet derived growth factor-BB-induced vascular smooth muscle cells phenotype modulation through STAT3 signaling pathway. Biochem. Biophys. Res. Commun..

[25] Gannon NP, Vaughan RA, Garcia-Smith R, Bisoffi M, Trujillo KA (2015). Effects of the exercise-inducible myokine irisin on malignant and non-malignant breast epithelial cell behavior *in vitro*. Int. J. Cancer.

[26] Aydin S, Kuloglu T, Ozercan M, Albayrak S, Aydin S, Bakal U, Yilmaz M, Kalayci M, Yardim M, Sarac M (2016). Irisin immunohistochemistry in gastrointestinal system cancers. Biotech. Histochem..

[27] Provatopoulou X, Georgiou GP, Kalogera E, Kalles V, Matiatou MA, Papapanagiotou I, Sagkriotis A, Zografos GC, Gounaris A (2015). Serum irisin levels are lower in patients with breast cancer: association with disease diagnosis and tumor characteristics. BMC Cancer.

[28] Us Altay D, Keha EE, Ozer Yaman S, Ince I, Alver A, Erdogan B, Canpolat S, Cobanoglu U, Mentese A (2016). Investigation of the expression of irisin and some cachectic factors in mice with experimentally induced gastric cancer. QJM Int. J. Med..

[29] Kuloglu T, Celik O, Aydin S, Ozercan IH, Acet M, Aydin Y, Artas G, Turk A, Yardim M, Ozan G (2016). Irisin immunostaining characteristics of breast and ovarian cancer cells. Cell. Mol. Biol..

[30] Yu L, Chen X, Sun X, Wang L, Chen S (2017). The glycolytic switch in tumors: how many players are involved?. J. Cancer.

[31] Singh D, Arora R, Kaur P, Singh B, Mannan R, Arora S (2017). Overexpression of hypoxia-inducible factor and metabolic pathways: possible targets of cancer. Cell Biosci..

[32] Nagao A, Kobayashi M, Koyasu S, Chow CC, Harada H (2019). HIF-1-dependent reprogramming of glucose metabolic pathway of cancer cells and its therapeutic significance. Int. J. Mol. Sci..

[33] Masoud GN, Li W (2015). HIF-1α pathway: role, regulation and intervention for cancer therapy. Acta Pharm. Sin. B.

[34] Casey, S. C., Amedei, A., Aquilano, K., Azmi, A. S., Benencia, F., Bhakta, D., Bilsland, A. E., Boosani, C. S., Chen, S. & Ciriolo, M. R. Cancer prevention and therapy through the modulation of the tumor microenvironment, in *Seminars in Cancer Biology*. S199–S223 (Elsevier, 2015). 10.1016/j.semcancer.2015.02.007 PMC4930000 25865775

[35] Feitelson, M. A., Arzumanyan, A., Kulathinal, R. J., Blain, S. W., Holcombe, R. F., Mahajna, J., Marino, M., Martinez-Chantar, M. L., Nawroth R. & Sanchez-Garcia, I. Sustained proliferation in cancer: mechanisms and novel therapeutic targets, in *Seminars in Cancer Biology*. S25–S54 (Elsevier, 2015). 10.1016/j.semcancer.2015.02.006 PMC4898971 25892662

[36] Seeber LM, Horrée N, Vooijs MA, Heintz APM, van der Wall E, Verheijen RH, Van Diest PJ (2011). The role of hypoxia inducible factor-1alpha in gynecological cancer. Crit. Rev. Oncol./Hematol..

[37] McCullough DJ, Nguyen LM-D, Siemann DW, Behnke BJ (2013). Effects of exercise training on tumor hypoxia and vascular function in the rodent preclinical orthotopic prostate cancer model. J. Appl. Physiol..

[38] Pei H, Yang Y, Cui L, Yang J, Li X, Yang Y, Duan H (2016). Bisdemethoxycurcumin inhibits ovarian cancer via reducing oxidative stress mediated MMPs expressions. Sci. Rep..

[39] Kong G, Jiang Y, Sun X, Cao Z, Zhang G, Zhao Z, Zhao Y, Yu Q, Cheng G (2017). Irisin reverses the IL-6 induced epithelial-mesenchymal transition in osteosarcoma cell migration and invasion through the STAT3/Snail signaling pathway. Oncol. Rep..

[40] da Cunha ND, Durigan RdCM, Tibana RA, Durigan JLQ, Navalta JW, Prestes J (2015). The response of matrix metalloproteinase-9 and-2 to exercise. Sports Med..

[41] Chen C-L, Chu J-S, Su W-C, Huang S-C, Lee W-Y (2010). Hypoxia and metabolic phenotypes during breast carcinogenesis: expression of HIF-1α, GLUT1, and CAIX. Virchows Arch..

[42] Semenza GL (2010). Defining the role of hypoxia-inducible factor 1 in cancer biology and therapeutics. Oncogene.

[43] Miyazawa M, Yasuda M, Fujita M, Hirasawa T, Kajiwara H, Hirabayashi K, Ogane N, Shimizu M, Asanuma H, Murakami M (2009). Association of hypoxia-inducible factor-1 expression with histology in epithelial ovarian tumors: a quantitative analysis of HIF-1. Arch. Gynecol. Obstet..

[44] Shimogai R, Kigawa J, Itamochi H, Iba T, Kanamori Y, Oishi T, Shimada M, Sato S, Kawaguchi W, Terakawa N (2008). Expression of hypoxia-inducible factor 1α gene affects the outcome in patients with ovarian cancer. Int. J. Gynecol. Cancer.

[45] Jin Y, Wang H, Liang X, Ma J, Wang Y (2014). Pathological and prognostic significance of hypoxia-inducible factor 1α expression in epithelial ovarian cancer: a meta-analysis. Tumor Biol..

[46] Majmundar AJ, Wong WJ, Simon MC (2010). Hypoxia-inducible factors and the response to hypoxic stress. Mol. Cell.

[47] Miyazaki Y, Hara A, Kato K, Oyama T, Yamada Y, Mori H, Shibata T (2008). The effect of hypoxic microenvironment on matrix metalloproteinase expression in xenografts of human oral squamous cell carcinoma. Int. J. Oncol..

[48] Bertout JA, Patel SA, Simon MC (2008). The impact of O_2_ availability on human cancer. Nat. Rev. Cancer.

[49] Cairns RA, Harris IS, Mak TW (2011). Regulation of cancer cell metabolism. Nat. Rev. Cancer.

[50] Qiao Q, Nozaki Y, Sakoe K, Komatsu N, Kirito K (2010). NF-κB mediates aberrant activation of HIF-1 in malignant lymphoma. Exp. Hematol..

[51] Valvona CJ, Fillmore HL, Nunn PB, Pilkington GJ (2016). The regulation and function of lactate dehydrogenase a: therapeutic potential in brain tumor. Brain Pathol..

[52] Brenner DR, Ruan Y, Adams SC, Courneya KS, Friedenreich CM (2019). The impact of exercise on growth factors (VEGF and FGF2): results from a 12-month randomized intervention trial. Eur. Rev. Aging Phys. Act..

[53] Hu K, Babapoor-Farrokhran S, Rodrigues M, Deshpande M, Puchner B, Kashiwabuchi F, Hassan SJ, Asnaghi L, Handa JT, Merbs S (2016). Hypoxia-inducible factor 1 upregulation of both VEGF and ANGPTL4 is required to promote the angiogenic phenotype in uveal melanoma. Oncotarget.

