# Supplementary material for: Effects of the exercise-inducible myokine irisin on proliferation and malignant properties of ovarian cancer cells through the HIF-1 α signaling pathway
Source: Sci Rep. 2023 Jan 4;13:170. doi: 10.1038/s41598-022-26700-2 (PMC9813258; doi:10.1038/s41598-022-26700-2)
Supplement: Supplementary file 1 — Supplementary Information. [file 41598_2022_26700_MOESM1_ESM.docx]

**A**

**B**

**C**

**D**

**E**


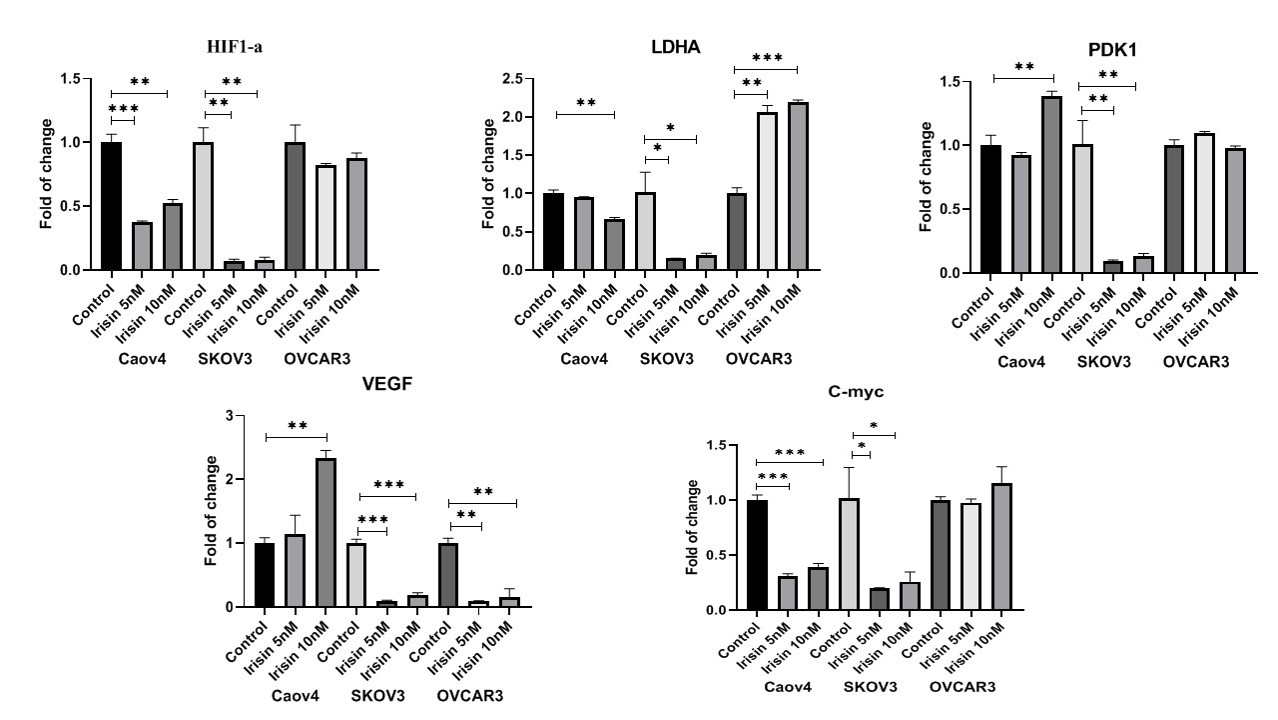


Effect of Irisin on HIF-1α signaling pathway genes that were normalized with GAPDH 48h after treatment with 5nM and 10nM of irisin. Data expressed as mean ± standard deviation; **P* ≤ 0.05 ***P* ≤ 0.01; ****P* ≤ 0.001 compared to control.
